# Poisoning from the Pollen of the Common Yellow Tiger Lily

**Published:** 1862-10

**Authors:** 


### POISONING FROM THE YELLOW TIGER LILY.

Oct. 27th.—*Case of poisoning from the Pollen of the common yellow Tiger Lily.*—Dr. Jeffries Wyman read the following report of a case by Dr. R. T. Warren, of Waltham, Mass.

"Mrs. B. was making a call at a neighbor's, having with her a little daughter, 4 years old. The child was 'perfectly well,' the mother said, and had been so. It played with another little girl, and did not go out of the room during the call. The little girl came to Mrs. B., requesting her to go and see Fanny, the name of the child. Mrs. B. went, and found Fanny rubbing her nose very violently. Soon there was a profuse discharge of mucus from the nose, colored yellow. The mother questioned the child, and ascertained that she had reached her hand out of the window, taken an anther from a tiger lily, and passed it into the right nostril. The child pointed out the lily, and the mother found just one anther missing. Mrs. B. was particular in her inquiries, and the child was positive in stating what she had done. Vomiting soon followed the discharge of mucus from the nose. This consisted at first of chyme, having no appearance of undigested food, and was followed by vomiting of mucus, colored yellow, the same as the discharge from the nose. The child then wanted to go to sleep. The mother took her home, and then sent for me. I saw her at 6, P. M., Wednesday, August 13, about an hour after the anther was passed into the nose. The child appeared sleepy, but was easily roused, and was intelligent. Vomiting of mucus, tinged yellow, occurred while I was present. The yellowness did not seem to be caused by bile. The symptoms did not seem at all alarming. Not aware that the tiger lily possessed any poisonous properties, I felt no anxiety, and went away, after prescribing remedies, requesting to be called if anything new occurred. I was sent for about 10, P.M., four hours afterwards. Evacuations of the bowels had occurred; at first of natural appearance, then followed discharges colored yellow, the same as the vomiting and the discharge from the nose, and at last bloody discharges. The vomiting had occurred occasionally, and this at last became bloody. The child was dull, sleepy, and languid. I prescribed astringents, opiates in the form of paregoric, and brandy and water, if the languor should increase. I saw her on Thursday morning. A dejection, quite bloody, occurred between 1 and 2 o'clock, A.M., and after that the dejections were checked. She was relieved of the vomiting. The child seemed languid, rather sleepy; no wandering. The eyes had a dull, reddish injection. At 4, P.M., same day, appearance of the child much the same as in the morning. The right nostril was nearly closed; membrane of both nostrils very pale. Some discharge of clear, thin mucus. Friday morning.—The child looked brighter. Same reddish injection of the eyes. No urine had been passed during the last twenty-four hours. Slight feverish symptoms. No delirium. 7, P.M., Friday.—No urine had been passed. Several dejections, dark colored, very offensive. Some fever during the day, slight delirium and startings. Some nausea. Was called to her about 1 o'clock, Saturday morning. Shortly before she had a large, dark-colored, very offensive discharge, and immediately began to sink. She died a little before 4 o'clock, about fifty-nine hours after passing the anther into the nostril."—*Boston Journal.*

